# A Novel Robust Position Integration Optimization-Based Alignment Method for In-Flight Coarse Alignment

**DOI:** 10.3390/s24217000

**Published:** 2024-10-31

**Authors:** Xiaoge Ning, Jixun Huang, Jianxun Li

**Affiliations:** 1 Department of Automation, School of Electronic Information and Electrical Engineering, Shanghai Jiao Tong University, Shanghai 200240, China; nxg01@126.com; 2 Beijing Aerospace Times Optical-Electronic Co., Ltd., Beijing 100094, China; 3 Beijing Institute of Aerospace Control Devices, Beijing 100039, China

**Keywords:** in-flight alignment, SINS, OBA method, robust position integration formula

## Abstract

In-flight alignment is a critical milestone for inertial navigation system/global navigation satellite system (INS/GNSS) applications in unmanned aerial vehicles (UAVs). The traditional position integration formula for in-flight coarse alignment requires the GNSS velocity data to be valid throughout the alignment period, which greatly limits the engineering applicability of the method. In this paper, a new robust position integration optimization-based alignment (OBA) method for in-flight coarse alignment is presented to solve the problem of in-flight alignment under a prolonged ineffective GNSS. In this methodology, to achieve a higher alignment accuracy in case the GNSS is not effective throughout the alignment period, the integration of GNSS velocity into the local-level navigation frame is replaced by the GNSS position in the Earth-centered, Earth-fixed frame, which avoids the need for complete GNSS velocity data. The simulation and flight test results show that the new robust position integration method proposed in this paper achieves higher stability and robustness than the conventional position integration OBA method and can achieve an alignment accuracy of 0.2° even when the GNSS is partially time-invalidated. Thus, this greatly extends the application of the OBA method for in-flight alignment.

## 1. Introduction

The accuracy of the initial alignment determines the performance of a strapdown navigation system (SINS) [1,2], and the purpose of the initial alignment is to obtain the initial attitude or attitude matrix since the initial position and velocity can be accurately acquired using a global navigation satellite system (GNSS).

To solve the alignment problem under a swaying base, Qin [3] proposed a self-alignment method based on the vector observations that are constructed with the gravitational apparent motion.

Yan [4] extended this approach to in-motion alignment aided by a GNSS/odometer, deriving a velocity integration algorithm. However, it is worth noting that the algorithm aided by a GNSS is not the most simplified.

During the same period, Wu [5,6] proposed an optimization-based alignment (OBA) method for in-flight alignment aided by a GNSS. The OBA method is based on an attitude matrix decomposition technique that decomposes the required attitude matrix into two time-varying attitude matrices and a constant attitude matrix. The time-varying attitude matrices are directly calculated using the attitude update procedure, and the constant attitude matrix is obtained based on constructed vector observations using Davenport’s q-method. Wu derived a velocity integration formula and position integration formula, and the velocity integration formula is more accurate than Yan’s method.

Subsequently, many variants of the OBA method based on velocity integration have been derived using different integration procedures and types of observation information to construct vector observations. To improve the rapidity of alignment, a backtracking scheme-based OBA method was proposed in [7,8], which calculates the stored data with forward and backward processes to improve the alignment accuracy without taking much extra time. To extend its application, OBA methods aided by ground velocity in the body frame provided by a Doppler Velocity Log or odometer were derived in [9,10,11,12]. To solve the problem of polar region alignment, an OBA method was proposed in [13] that avoids calculating singularity in a traditional navigational frame. To achieve the in-motion initial alignment of a low-cost SINS, dynamic OBA methods based on attitude estimation have been proposed [14,15], which can jointly estimate gyroscope bias and attitude errors.

However, relatively little research [16,17] has been conducted based on positional integration algorithms, and the position integration formula proposed by Wu [6] requires the GNSS velocity data to be valid throughout the alignment period, which is difficult to achieve in some application environments.

In this paper, a new robust position integration OBA method is proposed for in-flight alignment aided by a GNSS. To achieve greater alignment accuracy in case the GNSS is not effective throughout the alignment period, the integration of GNSS velocity into the local-level navigation frame is replaced by the GNSS position in the Earth-centered, Earth-fixed (ECEF) frame. The experimental results show that the new position integration OBA method proposed in this paper achieves better stability and robustness than the conventional position integration OBA method.

The remainder of this paper is organized as follows. Section 2 proposes a robust GNSS-aided position integration alignment method. Section 3 presents the simulations and experimental process and results. In Section 4, the conclusions are drawn.

## 2. Robust Position Integration Alignment Method Aided by GNSS

### 2.1. Traditional OBA Method

The navigation (attitude, velocity, and position) rate equations in the local-level navigation frame are, respectively, denoted as follows [1,2]:

(1) $${\dot{C}}_{b}^{n}=C_{b}^{n}(\omega_{nb}^{b}\times )$$

(2) $${\dot{v}}^{n}=C_{b}^{n}f^{b}-\left(2\omega_{ie}^{n}+\omega_{en}^{n}\right)\times v^{n}+g^{n}$$

(3) $$\dot{p}=R_{c}v^{n}$$

where $C_{b}^{n}$ denotes the attitude matrix from the body frame to the navigation frame, $v^{n}$ is the ground velocity in the navigation frame, $\omega_{ib}^{b}$ is the body angular rate in the body frame, $f^{b}$ is the specific force measured by accelerometers in the body frame, $\omega_{ie}^{n}$ is the Earth’s rotation rate expressed in the navigation frame, $\omega_{en}^{n}$ is the angular rate of the navigation frame with respect to the Earth frame expressed in the navigation frame, $\omega_{nb}^{b}=\omega_{ib}^{b}-C_{n}^{b}\omega_{in}^{n}$ is the body angular rate with respect to the navigation frame, and $g^{n}$ is the gravity vector. The 3 × 3 skew symmetric matrix (⋅×) is defined so that the cross-product satisfies a×b=(a×)b for two arbitrary vectors. The position $p={\left[\lambda \;\;L\;\;h\right]}^{T}$ is described by the angular orientation of the navigation frame relative to the Earth frame, commonly expressed as longitude λ , latitude L, and the height above the Earth’s surface h. $R_{c}$ is the local curvature matrix that is a function of the current position. All the quantities above are functions of time, and their time dependencies are omitted for brevity if not stated.

The alignment process starts from t = 0, and the measured velocity $v^{n}$ and position p over the time interval of interest [0, t] are obtained by the GNSS.

According to the chain rule [5] of the attitude matrix, the attitude matrix $C_{b}^{n}$ at any time satisfies

(4) $$C_{b}^{n}(t)=C_{n(0)}^{n(t)}C_{b}^{n}(0)C_{b(t)}^{b(0)}$$

where $C_{b(0)}^{b(t)}$ and $C_{n(0)}^{n(t)}$ can be determined through the attitude update procedure based on the angular rates $\omega_{in}^{n}$ and $\omega_{ib}^{b}$, respectively, as

(5) $$\left\{\begin{array}{c} {\dot{C}}_{b(t)}^{b(0)}=C_{b(t)}^{b(0)}(\omega_{ib}^{b}\times )\\ {\dot{C}}_{n(t)}^{n(0)}=C_{n(t)}^{n(0)}(\omega_{in}^{n}\times ). \end{array}\right.$$

Then, the core of the determining $C_{b}^{n}(t)$ is transformed to determine the constant matrix $C_{b}^{n}(0)$.

Substituting Equation (4) into Equation (2) and multiplying $C_{n(0)}^{n(t)}$ on both sides and reorganizing the terms yields

(6) $$C_{b}^{n}(0)C_{b(t)}^{b(0)}f^{b(t)}=C_{n(t)}^{n(0)}({\dot{v}}^{n(t)}+(2\omega_{ie}^{n(t)}+\omega_{en}^{n(t)})\times v^{n(t)}-g^{n(t)}).$$

Equation (6) forms the basis of the OBA method, which could be readily applied to the in-flight alignment problem discussed in this paper if velocity and position information were obtained from the GNSS.

It is required to differential the GNSS velocity in the calculation of ${\dot{v}}^{n(t)}$, so solving Equation (6) directly introduces a large noise error. Instead, we apply some integration algorithms, which are presented subsequently to obtain the matrix $C_{b}^{n}(0)$ from Equation (6). Later, matrix $C_{b}^{n}(t)$ can be obtained extremely readily from Equation (4).

Integrating Equation (6) on both sides over the time interval of interest yields the velocity integration formula as

(7) $$C_{b}^{n}(0)\alpha_{v}=\beta_{v}$$

in which

(8) $$\left\{\begin{array}{l} \alpha_{v}=\int_{0}^{t}C_{b(\tau )}^{b(0)}f^{b(\tau )}d\tau \\ \beta_{v}=\int_{0}^{t}C_{n(\tau )}^{n(0)}{\dot{v}}^{n(\tau )}d\tau +\int_{0}^{t}C_{n(\tau )}^{n(0)}(\omega_{ie}^{n(\tau )}+\omega_{in}^{n(\tau )})\times v^{n(\tau )}d\tau -\int_{0}^{t}C_{n(\tau )}^{n(0)}g^{n(\tau )}d\tau \\ \;\;\;\;=C_{n(\tau )}^{n(0)}v^{n(\tau )}\left|{\,}_{0}^{t}\right.-\int_{0}^{t}C_{n(\tau )}^{n(0)}\omega_{in}^{n(\tau )}\times v^{n(\tau )}d\tau \\ \;\;\;\;\;\;\;+\int_{0}^{t}C_{n(\tau )}^{n(0)}(\omega_{ie}^{n(\tau )}+\omega_{in}^{n(\tau )})\times v^{n(\tau )}d\tau -\int_{0}^{t}C_{n(\tau )}^{n(0)}g^{n(\tau )}d\tau \\ \;\;\;\;=C_{n(t)}^{n(0)}v^{n}(t)-v^{n}(0)+\int_{0}^{t}C_{n(\tau )}^{n(0)}\omega_{ie}^{n(\tau )}\times v^{n(\tau )}d\tau -\int_{0}^{t}C_{n(\tau )}^{n(0)}g^{n(\tau )}d\tau . \end{array}\right.$$

Then, we may use a two-vector method or multi-vector method (e.g., the Davenport q-method) to solve Equation (7), which is the velocity integration formula.

In aircraft flight conditions, the error of $\beta_{v}$ mainly derives from the initial and terminal GNSS velocity error, as the error in the last two terms is relatively small in magnitude.

Integrating Equation (6) on both sides twice over the time interval of interest and dividing the non-zero length of time on both sides yields the position integration formula as follows [6]:

(9) $$C_{b}^{n}(0)\alpha_{p}=\beta_{p}$$

in which

(10) $$\left\{\begin{array}{l} \alpha_{p}=\int_{0}^{t}\int_{0}^{\sigma }C_{b(\tau )}^{b(0)}f^{b(\tau )}d\tau d\sigma \\ \beta_{p}=\int_{0}^{t}C_{n(\tau )}^{n(0)}v^{n}(\tau )d\tau -tv^{n}(0)\\ \;\;\;\;\;\;\;+\int_{0}^{t}\int_{0}^{\sigma }C_{n(\tau )}^{n(0)}\omega_{ie}^{n(\tau )}\times v^{n(\tau )}d\tau d\sigma -\int_{0}^{t}\int_{0}^{\sigma }C_{n(\tau )}^{n(0)}g^{n(\tau )}d\tau d\sigma . \end{array}\right.$$

In the literature [6], the first term of $\beta_{p}$ is calculated as

(11) $$\begin{array}{l} \int_{0}^{t}C_{n(\tau )}^{n(0)}v^{n}(\tau )d\tau =\int_{0}^{t}C_{n(\tau )}^{n(0)}{\dot{r}}^{n}(\tau )d\tau \\ \;\;\;\;\;\;\;\;\;\;\;\;\;\;\;\;\;\;\;\;\;\;=C_{n(\tau )}^{n(0)}r^{n}(\tau )\left|{\,}_{0}^{t}-\right.\int_{0}^{t}C_{n(\tau )}^{n(0)}\omega_{in}^{n}\times r^{n}(\tau )d\tau \\ \;\;\;\;\;\;\;\;\;\;\;\;\;\;\;\;\;\;\;\;\;\;=C_{n(t)}^{n(0)}r^{n}(t)-\int_{0}^{t}C_{n(\tau )}^{n(0)}\omega_{in}^{n}\times r^{n}(\tau )d\tau \end{array}$$

where $r^{n}(t)=\int_{0}^{t}v^{n(\tau )}d\tau$ and $r^{n}$ satisfy

(12) $$\left\{\begin{array}{l} r^{n}(t)=\int_{0}^{t}v^{n(\tau )}d\tau =\int_{0}^{t}R_{c}^{-1}\dot{p}(\tau )d\tau \\ {\dot{r}}^{n}(t)=v^{n(t)}\\ r^{n}(0)=0. \end{array}\right.$$

Substituting this into Equation (10) yields the following:

(13) $$\begin{array}{l} \beta_{p}=C_{n(t)}^{n(0)}r^{n}(t)-\int_{0}^{t}C_{n(\tau )}^{n(0)}\omega_{in}^{n}\times r^{n}(\tau )d\tau -tv^{n}(0)\\ \;\;\;\;\;\;\;+\int_{0}^{t}\int_{0}^{\sigma }C_{n(\tau )}^{n(0)}\omega_{ie}^{n(\tau )}\times v^{n(\tau )}d\tau d\sigma -\int_{0}^{t}\int_{0}^{\sigma }C_{n(\tau )}^{n(0)}g^{n(\tau )}d\tau d\sigma . \end{array}$$

In aircraft flight conditions, the error of $\beta_{p}$ is mainly composed of the initial GNSS velocity error and the entire GNSS velocity error over the full integrating time, as the errors of the other terms are relatively small in magnitude.

Equations (9), (10) and (13) constitute the traditional position integration formula.

### 2.2. Novel Robust Position Integration Formula

Equation (2) is also known as the specific force equation in the navigation frame, and the specific force equation in the Earth frame is expressed as [2]

(14) $${\ddot{x}}^{e}=C_{b}^{e}f^{b}-2\omega_{ie}^{e}\times {\dot{x}}^{e}+g^{e}$$

where $x^{e}$ denotes the position of SINS in the Earth frame and ${\dot{x}}^{e}$ is the ground velocity in the Earth frame.

It is to be noted that the correlation between $x^{e}$ and $p={\left[\lambda \;\;L\;\;h\right]}^{T}$, and the correlation between $v^{n}$ and ${\dot{x}}^{e}$ is expressed as

(15) $$x^{e}=\left(\begin{array}{c} (N+h)\cos L\cos\lambda \\ (N+h)\cos L\sin\lambda \\ \left(N\left(1-e^{2}\right)+h\right)\sin L \end{array}\right)$$

(16) $$v^{n}=C_{e}^{n}{\dot{x}}^{e}$$

where N denotes the radius of curvature of the ellipsoid in the prime vertical plane, $e^{2}$ is the square of the first eccentricity of the ellipsoid, and $C_{e}^{n}$ is the matrix from the Earth frame to the navigation frame:

(17) $$C_{e}^{n}=\left[\begin{array}{ccc} -\sin\lambda & \cos\lambda & 0\\ -\sin L\cos\lambda & -\sin L\sin\lambda & \cos L\\ \cos L\cos\lambda & \cos L\sin\lambda & \sin L \end{array}\right].$$

**Figure 1:**
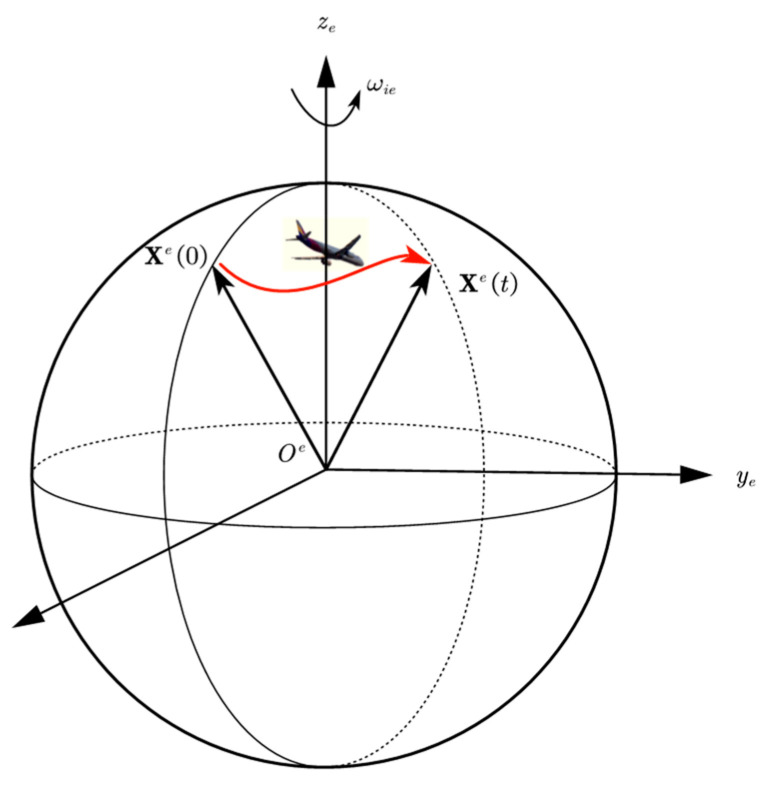
$x^{e}$ at different moments.

Substituting Equation (16), the first term of $\beta_{p}$ in Equation (10) is calculated as

(18) $$\begin{array}{l} \int_{0}^{t}C_{n(\tau )}^{n(0)}v^{n}(\tau )d\tau =\int_{0}^{t}C_{n(\tau )}^{n(0)}C_{e(\tau )}^{n(\tau )}{\dot{x}}^{e}(\tau )d\tau \\ \;\;\;\;\;\;\;\;\;\;\;\;\;\;\;\;\;\;\;\;\;\;=\int_{0}^{t}C_{e(\tau )}^{n(0)}{\dot{x}}^{e}(\tau )d\tau \\ \;\;\;\;\;\;\;\;\;\;\;\;\;\;\;\;\;\;\;\;\;\;=C_{e(\tau )}^{n(0)}x^{e}(\tau )\left|{\,}_{0}^{t}-\right.\int_{0}^{t}C_{e(\tau )}^{n(0)}\omega_{ie}^{e}\times x^{e}(\tau )d\tau \\ \;\;\;\;\;\;\;\;\;\;\;\;\;\;\;\;\;\;\;\;\;\;=C_{e(t)}^{n(0)}x^{e}(t)-C_{e(0)}^{n(0)}x^{e}(0)-\int_{0}^{t}C_{e(\tau )}^{n(0)}\omega_{ie}^{e}\times x^{e}(\tau )d\tau \end{array}$$

where $x^{e}$ can be obtained through Equation (15) using GNSS positions, $x^{e}(0)$ and $x^{e}(t)$ are shown in Figure 1.

Substituting Equation (18) into (10) yields

(19) $$\begin{array}{l} \beta_{p}=C_{e(t)}^{n(0)}x^{e}(t)-C_{e(0)}^{n(0)}x^{e}(0)-tv^{n}(0)-\int_{0}^{t}C_{e(\tau )}^{n(0)}\omega_{ie}^{e}\times x^{e}(\tau )d\tau \\ \;\;\;\;\;\;\;+\int_{0}^{t}\int_{0}^{\sigma }C_{n(\tau )}^{n(0)}\omega_{ie}^{n(\tau )}\times v^{n(\tau )}d\tau d\sigma -\int_{0}^{t}\int_{0}^{\sigma }C_{n(\tau )}^{n(0)}g^{n(\tau )}d\tau d\sigma . \end{array}$$

The error of $\beta_{p}$ in the new method is mainly constituted of the first three terms, as the errors of the other terms are relatively small in magnitude. The calculation method for the integral of the last three terms of Equation (19) can be found in the literature [6] and is not repeated here.

In Equation (13), the error of $\beta_{p}$ included the entire GNSS velocity error as all the GNSS velocity information was used to calculate the term $C_{n(t)}^{n(0)}r^{n}(t)$, i.e., the GNSS velocity data had to be valid throughout the alignment period. Once the GNSS velocity was unavailable, the algorithm produced a large error.

On the other hand, the error of $\beta_{p}$ in Equation (19) is mainly constituted of the initial GNSS velocity error and the initial and terminal GNSS position error, i.e., the method can work properly under a prolonged ineffective GNSS. In summary, the proposed new method would improve the alignment method’s robustness.

**Figure 2:**
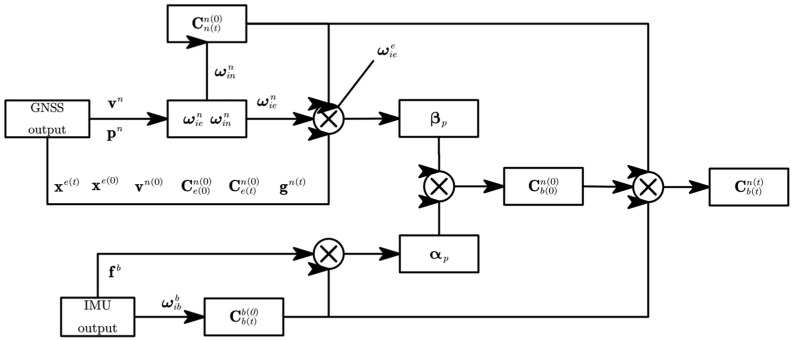
Diagram of the robust position integration formula method.

Equations (9), (10) and (19) constitute the robust position integration formula. A diagram of the proposed method is shown in Figure 2.

There are two types of solutions to Equations (6), (7) and (9). The first is called dual-vector attitude determination (tri-axial attitude determination, TRIAD [18]), and the second is called multi-vector attitude determination, which is the famous Wahba problem of attitude determination [19]. The Wahba problem has a series of solutions (i.e., the SVD method, Davenport q-method, FOAM method, ESOQ method, and ESOQ2 method) [20,21,22,23], and these solutions are essentially equal to each other [24]. In this paper, we employ the Davenport q-method method used in the literature [6].

(20) $$\begin{array}{l} \int_{0}^{t}C_{e(\tau )}^{n(0)}\omega_{ie}^{e}\times x^{e}(\tau )d\tau =\int_{0}^{t}C_{e(\tau )}^{n(0)}\omega_{ie}^{e}\times x^{e}(0)d\tau +\int_{0}^{t}C_{e(\tau )}^{n(0)}\omega_{ie}^{e}\times (x^{e}(\tau )-x^{e}(0))d\tau \\ \;\;\;\;\;\;\;\;\;\;\;\;\;\;\;\;\;\;\;\;\;\;\;\;\;\;\;\;=\int_{0}^{t}C_{i}^{n(0)}C_{e(\tau )}^{i}d\tau \omega_{ie}^{e}\times x^{e}(0)+\int_{0}^{t}C_{e(\tau )}^{n(0)}\omega_{ie}^{e}\times (x^{e}(\tau )-x^{e}(0))d\tau \\ \;\;\;\;\;\;\;\;\;\;\;\;\;\;\;\;\;\;\;\;\;\;\;\;\;\;\;\;=C_{i}^{n(0)}\int_{0}^{t}C_{e(\tau )}^{i}d\tau \omega_{ie}^{e}\times x^{e}(0)+\int_{0}^{t}C_{e(\tau )}^{n(0)}\omega_{ie}^{e}\times (x^{e}(\tau )-x^{e}(0))d\tau \end{array}$$

where

(21) $$\int_{0}^{t}C_{e(\tau )}^{i}d\tau =\int_{0}^{t}\left[\begin{array}{ccc} \cos\omega_{ie}^{\,}\tau & -\sin\omega_{ie}^{\,}\tau & 0\\ \sin\omega_{ie}^{\,}\tau & \cos\omega_{ie}^{\,}\tau & 0\\ 0 & 0 & 1 \end{array}\right]d\tau =\left[\begin{array}{ccc} \sin\omega_{ie}^{\,}t/\left|\omega_{ie}^{\,}\right| & -(1-\cos\omega_{ie}^{\,}t)/\left|\omega_{ie}^{\,}\right| & 0\\ (1-\cos\omega_{ie}^{\,}t)/\left|\omega_{ie}^{\,}\right| & \sin\omega_{ie}^{\,}t/\left|\omega_{ie}^{\,}\right| & 0\\ 0 & 0 & t \end{array}\right].$$

## 3. Simulations and Experiments

Simulations and experiments were conducted to verify and test the performance and advantages of the robust position integration formula (RPIF) proposed in this paper.

In these simulations and experiments, the RPIF method was compared with the traditional position integration formula (TPIF). The accuracy of these alignment methods was assessed using the difference between the true and calculated values of the attitude angle.

### 3.1. Simulation Results and Analysis

The simulation parameters and simulation environment were as follows:

The inertial device performance of the SINS was as follows: gyro drift 0.01°/h; random walk 0.001${}^{\circ}/\sqrt{h}$; accelerometer bias 50 μg; random noise 5 $\mu g/\sqrt{s}$. The SINS sampling rate was 100 Hz.

GNSS error was as follows: velocity noise 0.1 m/s (standard variance) and position noise 2 m (standard variance). The GNSS sampling rate was 10 Hz.

**Figure 3:**
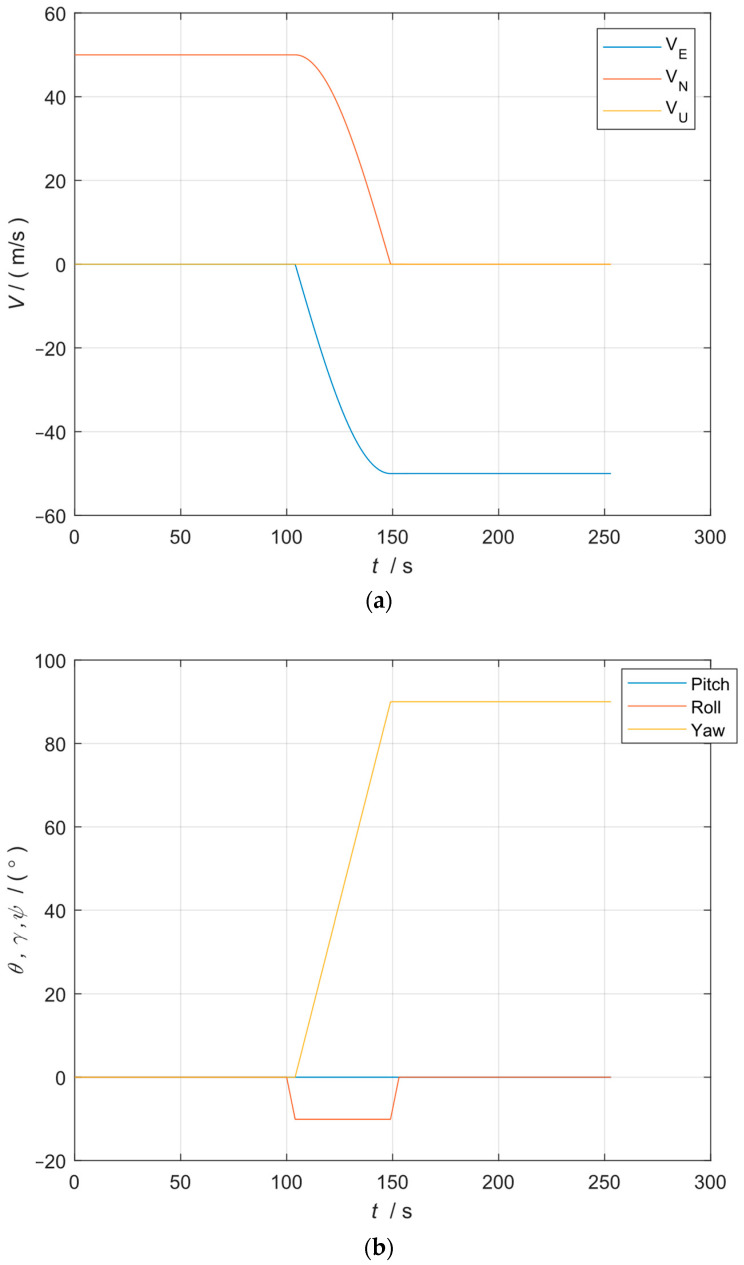

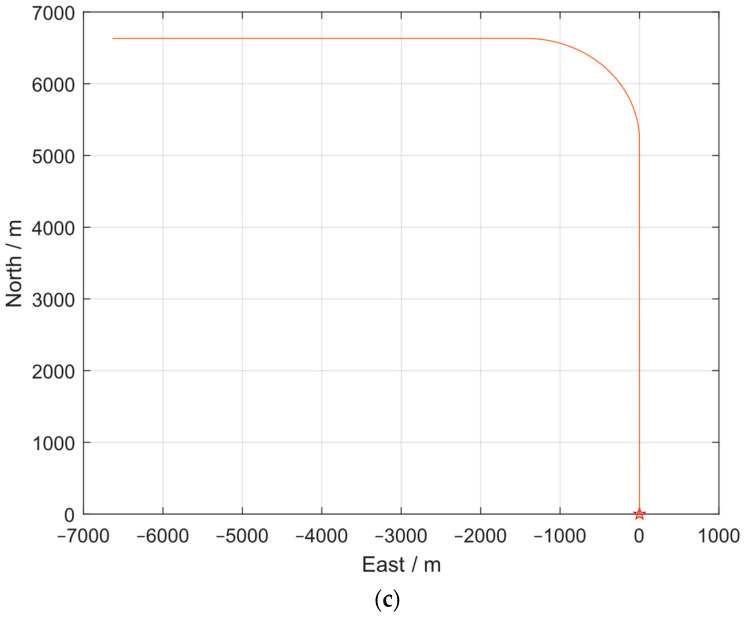
(**a**) Simulation velocity; (**b**) simulation attitude; (**c**) simulation trajectory position.

The initial position was 40° N, 116° E, and the initial attitude was 0°, 0°, 0°. The trajectory of the simulation is shown in Table 1 and Figure 3.

We conducted two sets of simulation experiments for the same working conditions. The GNSS data in the first set were valid throughout, while the GNSS data in the second set were invalid at 120~130 s during the turn.

**Figure 4:**
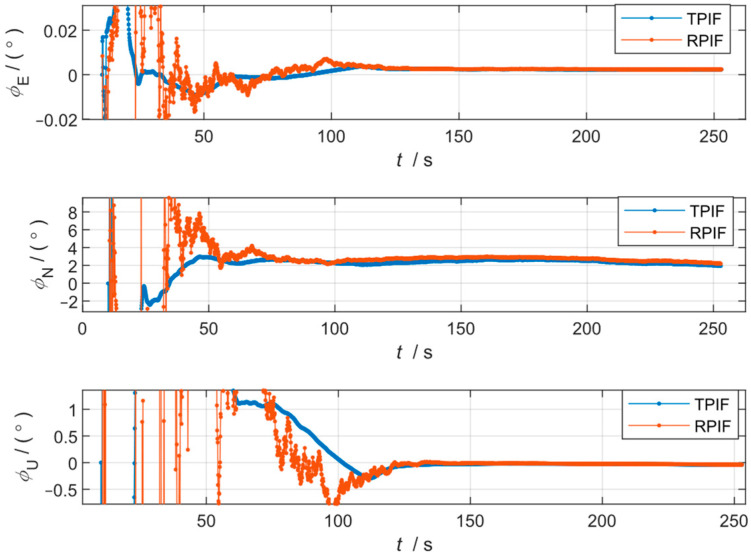
Curves of the alignment attitude error of the two methods for the first simulation condition.

**Figure 5:**
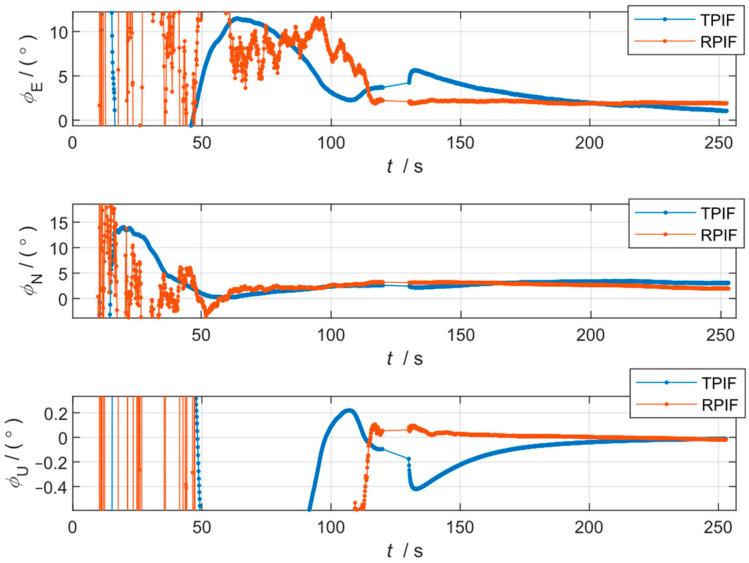
Curves of the alignment attitude error of the two methods for the second simulation condition.

The results of the first and second sets are shown in Figure 4 and Figure 5, respectively, and the misalignment angles at the end moment of the two simulations are shown in Table 2.

From Figure 4 and Table 2, it can be observed that the accuracy of the two methods is comparable when the GNSS data are valid throughout the working condition.

However, it can be seen from Figure 5 that under GNSS failure, the conventional method, TPIF, will produce a large mutation error, especially the azimuth, which presents a 0.4° jump error. Meanwhile, the attitude mutation of the RPIF method proposed in this paper is almost negligible. It should be noted that in Table 2, although the errors of the TPIF appear lower than those of the RPIF under the second simulation condition, the curve of the TPIF has, in fact, drifted, while the curve of the RPIF has stabilized. That is to say, the present method is more resistant to interference and at the same time has a stronger robustness.

It is worth noting that the fluctuations in errors of the proposed method, RPIF, are significantly larger than those of the conventional method, TPIF, during the initial phase of alignment. The reason for this is the fact that the errors in the proposed method, RPIF, include the position inaccuracy of the GNSS, while the errors in the traditional method mainly originated from the integration of the GNSS velocity inaccuracies. A position inaccuracy of 2 m is greater than the integral of a velocity white noise of 0.1 m/s.

### 3.2. Field Results and Analysis

We used flight data from a fiber-optic SINS on an unmanned aerial vehicle (UAV) for the validation assessment of the algorithm. The SINS/GNSS-integrated navigation results were used as the reference data for comparison. The SINS–GNSS lever arm was measured and largely eliminated.

The performance of the SINS was as follows: gyro drift 0.01°/h, random walk 0.001$\text{°}/\sqrt{h}$, accelerometer bias 20 μg, random noise 5 $\mu g/\sqrt{s}$. The SINS sampling rate was 100 Hz. The GNSS sampling rate was 10 Hz, the GNSS velocity noise was 0.1 m/s, and the position noise was 5 m.

**Figure 6:**
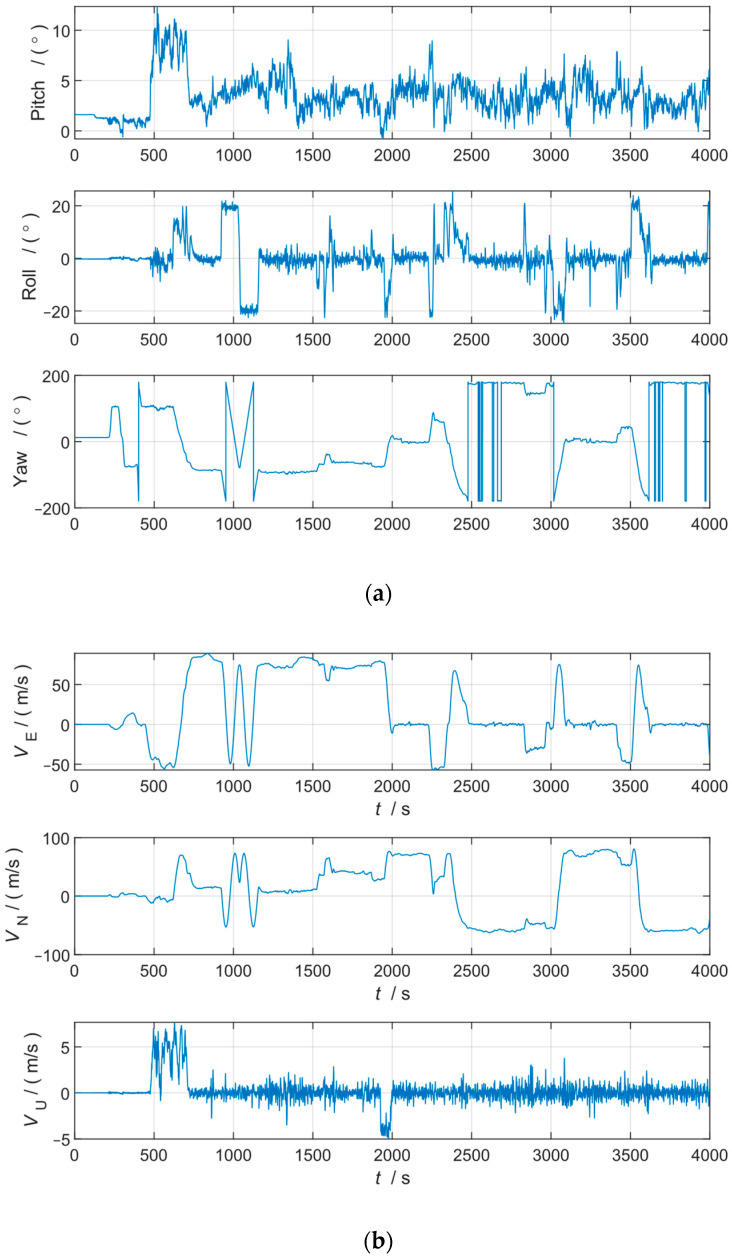

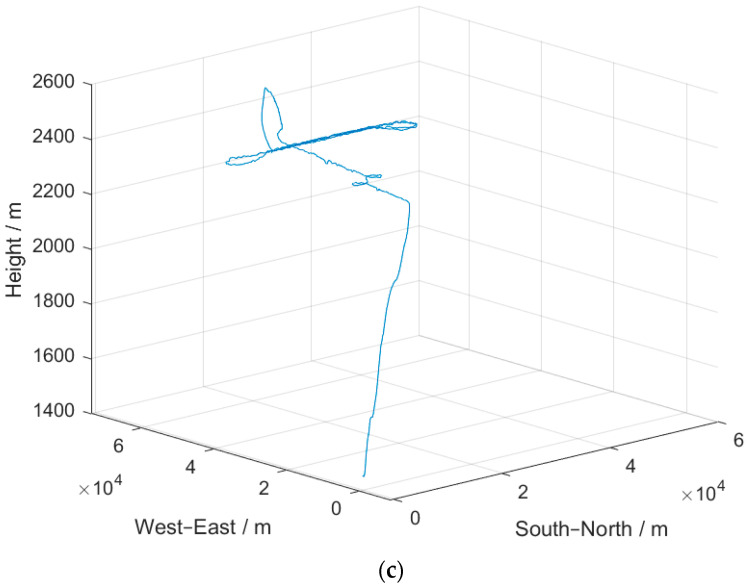
(**a**) Flight attitude; (**b**) flight velocity; (**c**) flight trajectory.

The UAV flight attitude, velocity, and trajectory are shown in Figure 6. We selected the turn segment data between 1850 s and 2200 s and invalidated the GNSS data between 1950 s and 2000 s to test the robustness of the algorithm under complex working conditions.

**Figure 7:**
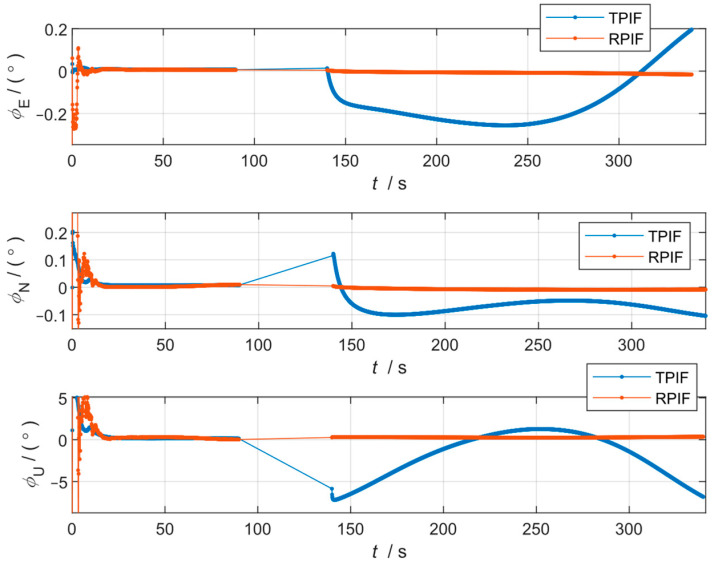
Curves of the alignment attitude error of the TPIF method and RPIF method flight data.

The alignment results of the TPIF method and RPIF method are shown in Figure 7 and Table 3.

As evident from Figure 7 and Table 3, the TPIF method not only suffers from great and abrupt attitude changes under extreme conditions when the GNSS is ineffective for a long period of time, but the final alignment result also becomes completely distorted. Nevertheless, the RPIF method proposed in this paper can still complete the in-flight alignment, and the alignment accuracy is better than 0.2°.

Under these conditions, the GNSS velocity was unavailable under maneuvering. The TPIF method integrated all the GNSS velocity information to calculate the term $r^{n}(t)$ in Equation (12) and was forced to integrate other velocity data under GNSS velocity invalidation; the algorithm then produced a large error, which seriously affected the results. In contrast, the RPIF method proposed in this paper was not affected as it did not require GNSS velocity data to be accurate at all moments in time.

Under this extreme condition, the conventional TPIF method is completely unusable, whereas the RPIF method maintains a considerable degree of accuracy. This fully demonstrates the robustness of the RPIF method proposed in this paper.

## 4. Conclusions

The traditional position integration formula for in-flight coarse alignment requires GNSS velocity data to be valid throughout the alignment period, which is difficult to achieve in some application environments. This has greatly limited the engineering applicability of the method.

In this paper, a new robust position integration OBA method for in-flight coarse alignment was presented to solve the problem of in-flight alignment under a prolonged ineffective GNSS. In this methodology, to achieve a higher alignment accuracy in cases where the GNSS is ineffective throughout the alignment period, the integration of GNSS velocity in the local-level navigation frame is replaced by the GNSS position in the ECEF frame, effectively avoiding the need for complete GNSS velocity data.

The experimental results show that the new robust position integration method proposed in this paper achieves better stability than the conventional position integration OBA method. This greatly extends the application of the OBA method for in-flight alignment.

## Figures and Tables

**Table 1:** The simulation trajectory, including three phases.

**No.**	**State**	**Duration**
1	Moving north with 50 m/s speed	100 s
2	Left turning	53 s
3	Moving west with 50 m/s speed	100 s

**Table 2:** The misalignment angle at the end moment of the two simulations.

**Alignment results under the first simulation condition**			
**Alignment methods**	**East misalignment (deg)**	**North misalignment (deg)**	**Upward misalignment (deg)**
TPIF	0.002	0.002	−0.041
RPIF	0.002	0.002	−0.038
**Alignment results under the second simulation condition**			
**Alignment methods**	**East misalignment (deg)**	**North misalignment (deg)**	**Upward misalignment (deg)**
TPIF	0.001	0.003	−0.013
RPIF	0.002	0.002	−0.019

**Table 3:** The misalignment angle at the end moment of in-flight alignment.

Alignment Methods	East Misalignment (deg)	North Misalignment (deg)	Upward Misalignment (deg)
TPIF	0.192	−0.106	−6.826
RPIF	0.027	−0.041	0.191

## Data Availability

The original contributions presented in the study are included in the article, further inquiries can be directed to the corresponding author.
